# Multifaceted Role of AMPK in Viral Infections

**DOI:** 10.3390/cells10051118

**Published:** 2021-05-06

**Authors:** Maimoona Shahid Bhutta, Elisa S. Gallo, Ronen Borenstein

**Affiliations:** 1 Department of Microbiology and Molecular Cell Biology, Eastern Virginia Medical School, Norfolk, VA 23507, USA; bhuttam@evms.edu; 2 Board-Certified Dermatologist and Independent Researcher, Norfolk, VA 23507, USA; esgallomd@hotmail.com

**Keywords:** AMPK, virus, COVID-19, catabolic process, anabolic processes, autophagy, apoptosis, lipid metabolism, fatty acid metabolism, mitochondrial homeostasis

## Abstract

Viral pathogens often exploit host cell regulatory and signaling pathways to ensure an optimal environment for growth and survival. Several studies have suggested that 5′-adenosine monophosphate-activated protein kinase (AMPK), an intracellular serine/threonine kinase, plays a significant role in the modulation of infection. Traditionally, AMPK is a key energy regulator of cell growth and proliferation, host autophagy, stress responses, metabolic reprogramming, mitochondrial homeostasis, fatty acid β-oxidation and host immune function. In this review, we highlight the modulation of host AMPK by various viruses under physiological conditions. These intracellular pathogens trigger metabolic changes altering AMPK signaling activity that then facilitates or inhibits viral replication. Considering the COVID-19 pandemic, understanding the regulation of AMPK signaling following infection can shed light on the development of more effective therapeutic strategies against viral infectious diseases.

## 1. Introduction

5′-adenosine monophosphate-activated protein kinase (AMPK) is an intracellular serine/threonine kinase that acts as a key metabolic regulator in maintaining cellular energy [1]. AMPK is activated by metabolic stress and acts to restore energy balance by changing cellular metabolism to generate energy through catabolic pathways such as involving glucose uptake, and by the inhibition of non-essential anabolic processes including lipid, protein and carbohydrate biosynthesis [2]. AMPK is also involved in autophagy, mitochondrial homeostasis, and mitophagy [3].

Due to its important role in the cell’s homeostasis, AMPK is an important cellular factor that many viruses utilize for replication, as it involves an energy-dependent process requiring high cellular ATP levels [4,5,6]. Viruses use AMPK to manipulate autophagy [7,8], fatty acid and lipid metabolism [5,9], glucose metabolism [10,11] and many other cellular processes.

In this review, we describe the various pathways and mechanisms by which viruses utilize or inhibit AMPK. We also summarize the AMPK signaling activity that facilitates or inhibits viral replication. Finally, we dedicate a special section for the role of AMPK in coronavirus infection.

## 2. AMPK Structure

**Figure 1:**
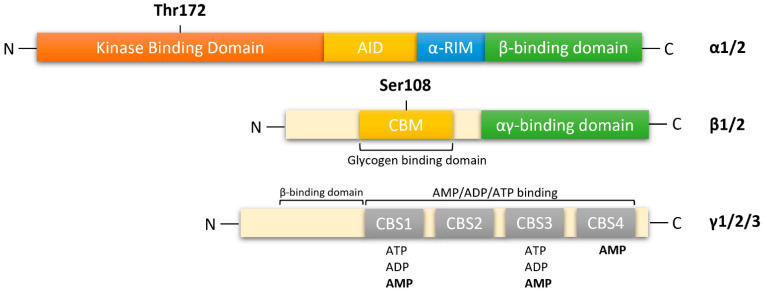
Functional domain of AMPK subunits. AMPK is a heterotrimeric complex composed of a catalytic α subunit(α1/2), regulatory β subunit (β1/2), and γ (γ1/2/3) subunit. AMPKα: kinase domain (KD) at the N-terminal contains Thr172, which is phosphorylated by upstream kinases; AID, auto-inhibitory domain; α-RIM: regulatory subunit interacting motif triggering conformational changes; β-subunit binding domain at the C-terminal. AMPKβ subunit: carbohydrate-binding module (CBM) near the N-terminal contains Ser108, which is important for the mechanism of action of some direct activators of AMPK; C-terminal domain containing the α-subunit-binding site and immediately followed by the domain for the γ-subunit interaction. αγ-binding domain: α-subunit-binding and γ-subunit interaction site at the C-terminal. AMPKγ subunit: cystathione-β-synthases (CBS) domain, which forms two Bateman domains containing four ATP/ADP/AMP-binding sites (CBS1–4).

AMPK is a heterotrimeric complex composed of a catalytic α subunit (two isoforms: α1/2), regulatory β subunit (two isoforms: β1/2) and γ subunit (three isoforms: γ1/2/3), which allows for the expression of 12 distinct complexes (Figure 1). The catalytic α subunit is characterized by serine/threonine kinase domains on the n-terminus and regulatory domain interactions occurring on the C-terminus [12,13,14]. The α1 isoform is widely expressed in all cells and accounts for 94% of the enzyme’s activity. Whereas, the α2 isoform is highly expressed in skeletal muscle, cardiac muscle, and liver [15]. In mammalian systems, activation of catalytic kinase domains occurs by the phosphorylation of a conserved threonine residue, Thr172, located in the activation loop [12]. In response to a decrease in cellular adenosine triphosphate (ATP) levels, the AMPK pathway is activated by three distinct mechanisms:

(1) Thr172 phosphorylation by upstream kinases [14,15,16,17,18];
(2) Inhibition of Thr172 dephosphorylation by protein phosphatases, PP2a or PP2c (mechanisms 1 and 2 require the presence of adenosine monophosphate (AMP) and adenosine diphosphate (ADP) [1];
(3) Allosteric activation of AMPK by AMP [19].

In response to decreasing AMP levels, auto inhibitory domain (AID) and regulatory subunit interacting motifs (α-RIM) on the C-terminal of the α subunit, maintain the α-kinase domains (α-KD) in an inactive conformation [13]. 

The β-subunits have myristoylated carbohydrate-binding molecules (CBM) in the N-terminus and interaction domains on the C-terminus [13,14]. In mammalian cells, modifications of AMPK occur cotranslationally by myristoylation of glycine residue 2 (Gly2) in the N-terminal of the β1-subunit [20,21]. This association allows β-subunits to form core complexes by linking the α C-terminus and γ N-terminus, forming an ST loop that contains phosphorylation sites for cAMP-dependent protein kinase (PKA) and serine/threonine protein kinases such as protein kinase B and glycogen synthase kinase (GSK) [14,22]. Hence, in response to AMP, myristoylation of the β-subunit is an essential requirement for AMPK signaling initiation [21]. The β1 isoform is more highly expressed in the liver than in skeletal muscle, whereas the β2 isoform is highly expressed in skeletal muscle [23]. 

The γ subunit contains four cystathionine-β-synthase (CBS) domains that create binding sites for adenine nucleotides, AMP, ADP and ATP (Figure 1). Thus, the γ-subunit plays an important role in AMPK regulation in response to cellular energy levels. CBS isoforms CBS1/CBS2 and CBS3/CBS4 assemble into complexes that generate four potential ligand-binding clefts in the center, which can be activated by AMP [13,14]. Regulatory site 3 in the CBS complex appears to be critical, as it binds to AMP at a greater affinity than ATP. AMP bound on regulatory site 3 facilitates interactions between α and γ subunits, leading to a compact conformational change in structure [14,24]. Conserved α-regulatory subunit interacting motif (α-RIM) within the α-linker interacts with the AMPK-γ subunit when AMP is bound at CBS3 [13]. Interestingly, phosphorylation of Thr172 by upstream kinases and simultaneous allosteric activation of AMPK (by binding of AMP to CBS domains) increases kinetic activity by ≥1000-fold [12,14,25]. As ATP displaces AMP on site 3, α-linkers are released from γ-subunits, reversing the conformational change. The regulatory γ subunit plays a vital role in activating the catalytic α subunit and the formation of heterotrimeric complexes [13,25]. The γ isoforms have the greatest variability in structure. The γ1 isoform (331 residues) is widely expressed in cells and tissues, and it is activated up to 3-fold [26]. Expression of γ2 (569 residues) is restricted to the brain, placenta, skeletal and cardiac muscle, where activation by AMP leads to a 3-fold increase. The γ3 isoform (489 residues) is restricted to skeletal muscle [26]. 

## 3. Regulation of AMPK Pathway

### 3.1. Activation of AMPK

Many cellular stresses can lead to AMPK activation, such as physiological changes in AMP/ATP and ADP/ATP ratios resulting from stressors such as nutrient starvation [27], hypoxia [28], prolonged exercise [29,30], pharmacological agents [31,32,33] and disease states [34,35]. As mentioned previously, an increase in AMPK activity is allosterically regulated by AMP binding to the AMPKγ subunit. This enhances phosphorylation of Thr172, causing a conformational change of the active loop, thus preventing dephosphorylation of Thr172 by phosphatases in the kinase domain activation loop [36,37,38].

**Figure 2:**
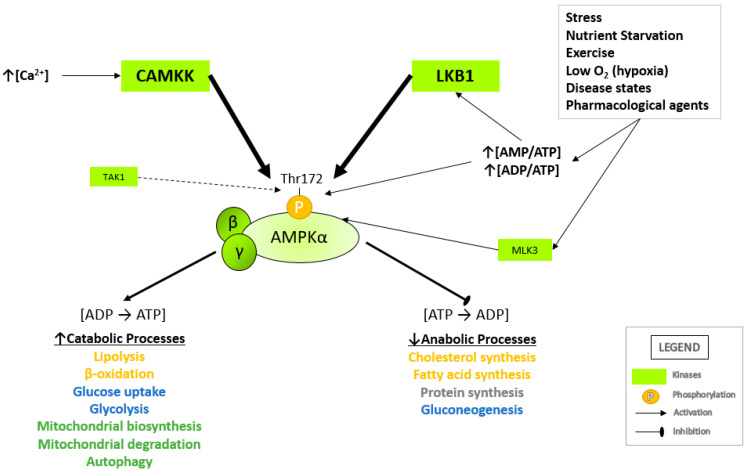
Activation of AMPK by upstream kinases. AMPK is activated following the phosphorylation of Thr172 on the catalytic α-subunit by upstream kinases in response to shifting adenosine nucleotide levels, cytosolic calcium levels and external stressors. Following activation, AMPK regulates anabolic, ATP-consuming pathways and catabolic, ATP-generating pathways. A summary of the physiological roles of AMPK is listed above (arrow indicates activation/increase; bar indicates inhibition/decrease). LKB1, liver kinase B1; CAMKK2, calcium/calmodulin-dependent kinase kinase 2; TAK1, transforming growth factor-β-activated kinase 1; MLK3, mixed lineage kinase 3.

Upstream kinases known to phosphorylate AMPK at Thr172 are the serine-threonine liver kinase B1 (LKB1), Ca^2+^/calmodulin-dependent kinase kinases (CaMKKs) [12,29] transforming growth factor-β-activated kinase 1 (TAK1) [17] and mixed lineage kinase 3 (MLK3; Figure 2) [14,17,18]. LKB1 forms a heterotrimeric active complex with sterile-20-related adaptor (STRAD) and mouse protein 25 (MO25) and directly mediates the phosphorylation of Thr172 following an increase in cellular ADP/ATP and AMP/ATP ratios. In conjunction with increasing AMP or ADP levels, Thr172 can also be phosphorylated by CAMKK2 (CAMKK-β) in response to increasing intracellular Ca^2+^ levels [39,40]. TAK1 is a serine/threonine protein kinase of the mitogen-activated protein kinase kinase kinase (MAP3K) family. TAK1, which is activated by inflammatory cytokines such as interleukin 1 (IL-1), tumor necrosis factor (TNF) and TGF-β receptors, toll-like receptors (TLR), CD40 and the B cell receptor, has also been reported to phosphorylate Thr172 to activate AMPK [15,37]. MLK3 is a serine/threonine protein kinase and serves as a common upstream kinase of AMPK and c-Jun N-terminal kinase (JNK) pathway [36]. Once activated, AMPK restores ATP levels by activating catabolic pathways that regenerate ATP while inhibiting anabolic pathways that consume ATP [41] (Figure 2).

### 3.2. Role of AMPK in Metabolism

#### 3.2.1. Carbohydrate Metabolism

**Figure 3:**
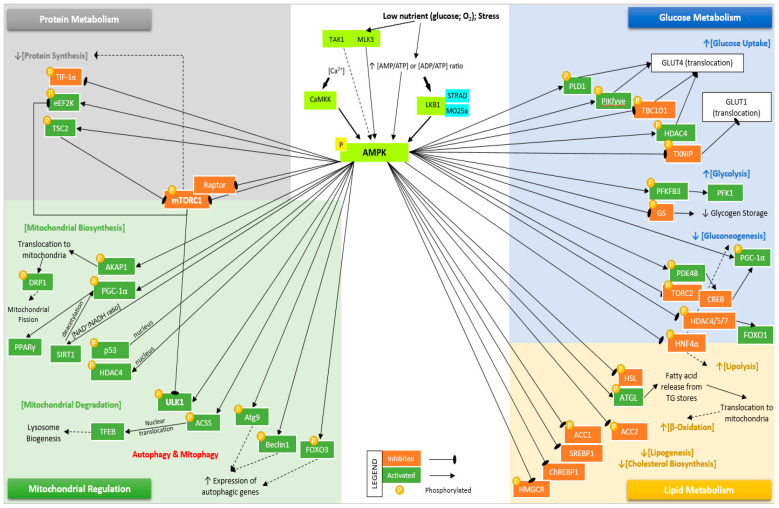
Summary of differentially expressed genes involved in the AMPK signaling pathway. Activation of AMPK occurs following phosphorylation of Thr172 (not shown) by LKB1, CaMKK, TAK1, and MLK3. Activated AMPK regulates glucose metabolism by increasing glucose uptake via translocation of GLUT4 by phosphorylating/inhibiting TBC1D1 and phosphorylating/activating PIKfyve, HDAC4, and PLD1. AMPK-mediated translocation of GLUT1 occurs following phosphorylation/inhibition of TXNIP. Glycolysis is stimulated via activation of PFKFB3, and glycogen storage is reduced by inhibition of GS. Inhibition of gluconeogenesis occurs following phosphorylation/inhibition of PDE4B, TORC2, HDAC4/5/6, and HNF4α. AMPK regulates lipid metabolism by phosphorylating/inhibiting HSL, HNF4α, and activating ATGL to increase lipolysis. An increase in β-oxidation occurs by phosphorylation of ACC2 and reducing fatty acid synthesis by phosphorylation of ACC1. AMPK decreases lipid and sterol synthesis by phosphorylating/inhibiting SREBP1, ChREBP, and HMGCR. AMPK inhibits protein synthesis by phosphorylating/inhibiting TIF-1α, mTORC1, and RAPTOR, and phosphorylating/activating eEF2K and TSC2, which inhibit mTORC1. Lastly, mitochondrial functions are regulated by activated AMPK by activating mitochondrial biosynthesis. AMPK phosphorylates/activates AKAP1, DRP1, PGC-1α, SIRT1, p53, and HDAC4. AMPK activates mitophagy and autophagy pathways by phosphorylating/activating ULK1, ACSS, Atg9, Beclin1, and FOXO3.

Activated AMPK promotes catabolic pathways with respect to glucose metabolism, such as glucose uptake and glycolysis, and inhibits gluconeogenesis activation (Figure 3). AMPK regulates glucose uptake by translocation of glucose transporter 4 (GLUT4) and glucose transporter 1 (GLUT1) by phosphorylating and inhibiting RAB GTPase-activating protein (GAP), TBC1 domain family member 1 (TBC1D1) [42] and thioredoxin-interacting protein (TXNIP) [43], respectively. Uptake of glucose is also promoted by AMPK phosphorylation of phosphoinositide phosphate kinase, phosphatidylinositol 3-phosphate 5-kinase (PIKfyve) and phospholipase D1 (PLD1), which regulates GLUT4 translocation, thus indirectly increasing glucose uptake [44,45]. AMPK phosphorylates and activates histone deacetylase 4 (HDAC4), enhancing GLUT4 expression [46]. AMPK stimulates glycolysis through the activation and phosphorylation of 6-phosphofructo-2-kinase/fructose-2,6-bisphosphatase 3 (PFKFB3), which affects the activity of 6-phosphofructo-1-kinase (PFK1) [47]. Glycogen synthase (GS) is inhibited following phosphorylation by AMPK, which reduces glycogen storage. However, the accumulation of glucose-6-phosphate (G6P), a precursor to UDP-glucose, can override the inhibitory effects caused by AMPK phosphorylation [48]. In mice models, activation of hepatic AMPK causes phosphorylation of phosphodiesterase 4B (PDE4B), which inhibits the glucagon-stimulated increase of cAMP and PKA signaling [49].

AMPK inhibits the transcriptional induction of gluconeogenesis by phosphorylating cyclic-AMP-regulated transcriptional coactivator 2 (CRTC2) [50] and class IIA histone deacetylases (HDAC4/5/6) [51], which are coactivators of the cAMP response element-binding protein (CREB) and forkhead box protein O (FOXO) pathways, respectively [3]. AMPK also inhibits gluconeogenesis by phosphorylating transducer of regulated CREB activity 2 (TORC2) complexed with CRE binding protein (CREB), thus preventing CREB-stimulated hepatic gluconeogenesis and fatty acid oxidation by inducing a nuclear hormone receptor coactivator, peroxisome-proliferation-activated receptor-γ coactivator-1 (PGC-1α) [50].

#### 3.2.2. Lipid Metabolism

AMPK reduces lipid storage through distinct pathways to promote fatty acid oxidation and suppression of lipogenesis and cholesterol biosynthesis (Figure 3). Previous studies show that phosphorylation by AMPK of rate-limiting enzymes regulating lipolysis activates adipose triglyceride lipase (ATGL) and inhibits hormone-sensitive lipase (HSL) [52]. Activation of ATGL promotes the release of fatty acids from triglyceride stores, which then translocate into the mitochondria for β-oxidation [53]. In response to cellular stress or exercise, AMPK promotes fatty acid oxidation with an increase in β-oxidation by phosphorylating acetyl-CoA carboxylase 2 (ACC2) and it reduces fatty acid synthesis by phosphorylating ACC1 [54,55]. AMPK also regulates lipid and sterol synthesis by phosphorylating and inhibiting 3-hydroxy-3-methyl-glutaryl-coA reductase (HMGCR), which converts 3-hydroxy-3-methylglutaryl coenzyme A (HMG-CoA) to mevalonate. This functions as the rate-limiting step of the mevalonate pathway [56]. Phosphorylation by AMPK leads to inhibition of key transcriptional regulators of lipid and glucose metabolism, sterol regulatory element-binding protein 1 (SREBP1) [57], hepatocyte nuclear factor-4α (HNF4α) [58] and carbohydrate-responsive element-binding protein (ChREBP) [59]. 

#### 3.2.3. Protein Metabolism

Inhibition of protein synthesis by AMPK is mediated by the inhibition of the mechanistic target of rapamycin complex 1 (mTORC1), which plays a central role in protein translation and cell growth (Figure 3). In energy shortage conditions, AMPK activation inhibits mTORC1 activity by two independent mechanisms: activation through phosphorylation of tuberous sclerosis complex 2 (TSC2), a negative regulator of mTORC [60], and inhibition through phosphorylation of regulatory-associated protein of mTOR (Raptor) [61,62]. AMPK inhibits ribosomal RNA synthesis by phosphorylating and inhibiting the RNA-polymerase I-associated transcription initiation factor IA (TIF-IA) at Ser-635 [63] and inhibits protein elongation by phosphorylating and activating eukaryotic elongation factor 2 kinase (eEF2K) [64]. eEF2K is also directly phosphorylated by mTORC1, thus establishing cross-talk between distinct pathways controlling cell growth and metabolism [65].

#### 3.2.4. Autophagy and Mitochondrial Homeostasis

By regulating mitochondrial functions, AMPK can control mitochondrial biogenesis, degradation (mitophagy), and autophagy (Figure 3). In response to metabolic stress, AMPK phosphorylates and activates a key regulator of mitochondrial fusion, A-kinase anchor protein 1 (AKAP1) [66]. AKAP1 is a mitochondrial scaffold protein that binds to mitochondrial-targeted protein kinase A (PKA), which then phosphorylates dynamin-related protein 1 (DRP1). DRP1 and AMPK-phosphorylated mitochondrial fission factor (MFF) play an essential role in initiating mitochondrial fission [67]. AMPK increases mitochondria biogenesis by phosphorylating and upregulating peroxisome proliferator-activated receptor-γ (PPARγ) and coactivator 1α (PGC1α). AMPK-mediated upregulation of PGC1α also involves activation of p53, sirtuin 1 (SIRT1) [68], and HDAC4 [69]. AMPK phosphorylates acetyl-CoA synthetase (ACSS), allowing nuclear translocation, which leads to the acetylation and activation of transcription factor EB (Tfeb), promoting lysosome biogenesis [3,70]. Following fission, AMPK promotes mitophagy by activating and phosphorylating unc-51-like autophagy activating kinase 1 (ULK1) at multiple sites, translocating ULK1 to mitochondria, and triggering the mitophagy cascade [71]. In nutrient starvation conditions, AMPK inhibits the mTORC1 complex, a key regulator of autophagy, through the activation of TSC2 and consequent inhibition of Rheb/mTORC1 signaling. AMPK-mediated inhibition of mTORC1 or RAPTOR prevents mTORC1-mediated inhibition of ULK1 [72]. Like mitophagy, autophagy is a critical stress response, which allows the cells to replace organelles, proteins, and other cellular components. AMPK also phosphorylates autophagy-related protein 9 (ATG9) [73], beclin 1 [74] and forkhead box protein O3 (FOXO3) [75], all of which in turn increase the expression of autophagy genes (ATG) involved in autophagosome biogenesis and maturation. AMPK was also reported to increases autophagic flux by contributing to autophagosome maturation and autolysosome fusion [76]. Thus, in response to stress, AMPK coordinates mitochondrial fission, mitophagy, and regulates autophagy in cells.

## 4. Interaction between Viruses and AMPK Pathways

### 4.1. Modulating AMPK through Catabolic and Anabolic Processes

#### 4.1.1. Modulation of Autophagy

Activation or inhibition of AMPK-related processes plays an important role in the survival of viruses (Table 1). Hepatitis B virus (HBV), a member of the Hepadnaviridae family of viruses, is a partially double-stranded DNA virus. HBV is the causative agent of hepatitis B, an infectious disease that affects the liver. In HepG2.2.15 cells that stably express HBV, PRKAA (a catalytic subunit of AMPK) is activated in response to HBV-induced oxidative stress, which in turn decreases HBV replication through the promotion of autophagic degradation [7]. In addition, p70 ribosomal S6 kinase (S6K1), a serine/threonine protein kinase, inhibits HBV replication through inhibition of the AMPK-ULK1 pathway and disruption of the acetylation modification of lysine 27 on histone H3 (H3K27) [77]. Furthermore, a high glucose concentration, activating the mTOR pathway, results in reduced HBV replication, while a low glucose concentration promotes HBV replication by stimulating the AMPK/mTOR-ULK1-autophagy axis [78].

Human cytomegalovirus (HCMV) is a beta herpesvirus with a 230-kb double-stranded DNA genome encoding over 200 proteins. The HCMV genome consists of unique sequences flanked by two sets of inverted repeats: internal repeat short (IRS) protein and terminal repeat short (TRS) protein. TRS1 and IRS1 are identified as viral tegument proteins in the infected cell cytoplasm and nucleus [79]. Mouna et al. demonstrated that TRS1 and IRS1 inhibit autophagy in starved and infected cells. In cell culture, inhibition of autophagy occurred following the interaction of IRS1 and TRS1 with the N-terminal domain of Beclin1. Co-expression of TRS1 and IRS1 blocked the formation of autophagosomes; however, the expression of either TRS1 or IRS1 partially controls autophagy [80]. In the search for HCMV inhibitors, digitoxin inhibited the α1 subunit pump-dependent AMPK activation and led to increased autophagy at a level that was able to inhibit HCMV [81]. Indeed, regulated autophagy plays an important role in HCMV life cycle and autophagy-initiating protein kinase ULK1 has been found to phosphorylate the HCMV tegument protein pp28 and to regulate virion release [8].

Hepatitis C virus (HCV) is a positive-stranded RNA virus with a 9.6-kb genome. HCV infection is a major cause of chronic liver disease. The insulin sensitizer metformin was used in HCV infected cells to activate AMPK; it induced the activation of type I interferon (IFNγ) signaling, which is an inducer of autophagy [82], and subsequently inhibited HCV replication [83].

On the other hand, in a recent study by Subramanian et al., it was shown that herpes simplex virus type 1 (HSV-1) replication depends on AMPK activity, but not on its function in autophagy. HSV-1 is a double-stranded DNA alpha herpesvirus of the Herpesviridae family. Subramanian et al. also reported that the interferon-inducible protein, TDRD7, inhibits AMPK and subsequently inhibits autophagy-independent HSV-1 replication [84]. Interestingly, Tudor domain-containing 7 (TDRD7) plays the same role in the infection of viruses from the Paramyxoviridae family, including human parainfluenza virus type 3, respiratory syncytial virus, and Sendai virus (SeV) [85].

Kaposi’s sarcoma associated herpesvirus (KSHV/HHV-8) is a gammaherpesvirus associated with human malignancies such as Kaposi’s sarcoma and primary effusion lymphoma. KSHV/HHV-8 has been reported to encode for proteins that mimic cellular orthologs, generating viral Bcl-2 (v-Bcl-2) and viral Fas-associated death domain-like interleukin-1β (IL-1β)-converting enzyme-like inhibitory protein (v-FLIP), since these proteins have a strong impact on the autophagic process. v-FLIP suppresses autophagy by preventing Atg3 from binding and processing microtubule-associated protein 1A/1B-light chain 3 (LC3) [86].

Respiratory syncytial virus (RSV), a member of the *Pneumovirus* genus in the Paramyxoviridae family, is an enveloped negative-stranded RNA virus. It is the main cause of acute lower respiratory tract infection in children. RSV infection induces autophagy through reactive oxygen species (ROS) generation and activation of the AMPK-mTOR signaling pathway and promotes viral replication [87].

Coxsackievirus B3 (CVB3) induces autophagy via AMPK/MEK/ERK and Ras/Raf/MEK/ERK signaling pathways in the host cells. While autophagy can clear a small portion of CVB3, the life cycle of CVB3 depends on autophagy, and therefore this process is essential for CVB3 pathogenesis. Indeed, CVB3 infection significantly increases the phosphorylation of AMPK [88]. The avian reovirus (ARV) is a segmented dsRNA virus of the family Reovirdae, and a known pathogen in poultry. ARV infection upregulates the phosphorylation of AMPK, and AMPK facilitates MKK 3/6, MAPK and p38 signaling, which are required for virus replication [89]. Chi et al. reported that the nonstructural protein 17 (NSP17) of ARV functions as an activator of autophagy by increasing levels of Beclin1 and LC3II. Modulation of NSP17-dependent autophagy in ARV occurs through the activation of p53/PTEN, AMPK, and dsRNA-dependent protein kinase (PKR) signaling [90].

AMPK-related autophagy is a mechanism utilized by many other viruses such as Epstein–Barr virus (EBV) [91], HIV [92], Newcastle disease virus (NDV)[93], porcine circovirus type 2 (PCV2) [94,95], avian reovirus (ARV)[90], influenza A [96], West Nile virus (WNV) [97], bluetongue virus (BTV) [98,99,100], duck enteritis virus (DEV) [101,102], rabies virus (RABV) [103,104] and swine fever virus (CSFV) [105].

#### 4.1.2. Modulation of Apoptosis

HBV causes hepatitis B, and chronic infection is one of the major risk factors for the development of hepatocellular carcinoma (HCC). In the HepG2 hepatoma cell line, HBV exerts an antiapoptotic effect by activating the AMPK/MnSOD signaling pathway, which is mediated by the HBV X protein [106]. In addition, HBx activation of both AMPK and mTORC1 in primary rat hepatocytes suggests that these activations work as a balancing mechanism to facilitate persistent HBV replication, and they could influence HCC development [107]. On the other hand, MicroRNA-1271 (miR-1271), a tumor suppressor in various cancers, promotes the activation of the AMPK signaling pathway by binding to CCNA1, resulting in inhibition of HBV-associated HCC. This occurs through inhibition of HBV-DNA replication, proliferation, migration and invasion, and acceleration of apoptosis [108].

KSHV infection of endothelial cells enhances angiogenesis, activates the PI3K/Akt/mTOR pathway and inactivates AMPK. This pathway confers a survival advantage and protects infected cells from apoptosis [109]. Furthermore, KSHV K1 protein promotes cell survival via its association with AMPKγ1 following exposure to stress. [110]. In addition, activated AMPK restricts KSHV lytic replication in primary human umbilical vein endothelial cells. Knockdown of the AMPKα1 and AMPK inhibitor, compound C, considerably enhances the expression of viral lytic genes and the production of infectious virions. Accordingly, the AMPK agonists, AICAR (5-aminoimidazole-4-carboxamide-1-β-D-ribofuranoside) and metformin, drastically inhibit the virus [111]. However, some viruses utilize activated AMPK to prevent apoptosis. In Zika virus infection of human foreskin fibroblasts, the virus causes a depletion in nucleotide triphosphate levels, leading to AMPK phosphorylation and caspase-mediated cell death [112].

#### 4.1.3. Regulation of Mitochondrial Function

In HSV-1 infection of mice neuronal cultures, HSV-1 modulates the AMPK/Sirt1 axis differentially during infection, interfering with proapoptotic signaling and regulating mitochondrial biogenesis. AMPK is downregulated during early infection; however, it recovers gradually. Furthermore, acetylated-p53, Sirt1 and p-AMPK translocate from the nucleus to the cytoplasm after 4 h of infection, and they accumulate in discrete foci in the perinuclear region [113]. Pretreatment of neurons with the natural activators of the AMPK/Sirt1 axis, resveratrol and quercetin, significantly increase the viability of infected neurons, reducing the viral titer and expression of viral genes [114]. 

In human immunodeficiency virus 1 (HIV-1) infection, HIV-1 Tat inhibits the AMPK signaling pathway through the NAD+/SIRT1 pathway and induces HIV-1 LTR transactivation [115]. In addition, MiR-217 is involved in Tat-induced HIV-1 LTR transactivation by downregulation of SIRT1 [116]. 

In the same venue, HCV induces hepatic metabolism disorders through downregulation of the SIRT1-AMPK signaling pathway [117]. 

The vesicular stomatitis virus (VSV) is an enveloped, negative-sense RNA virus of the family Rhabdoviridae, which infects a wide variety of mammalian and insect cells and can cause an influenza-like illness in humans. VSV infection targets AMPK, miR-33/33* specifically to prevent the mitochondrial adaptor, mitochondrial antiviral-signaling protein (MAVS) from forming activated aggregates and causing repressed antiviral innate immunity. VSV infection results in decreased expression of miR-33/33* in macrophages, leading to activation of AMPK and MAVS, promoting an antiviral innate immune response [118]. On the other hand, in SeV infection, mitochondrial fission factor (Mff) is phosphorylated by AMPK, leading to the disorganization of clusters of MAVS and repression of the acute antiviral response [119].

Epstein–Barr virus latent membrane protein 1 (EBV-LMP1) is a suppressor of the DNA damage response through DNA-PK/AMPK signaling, and promotes radioresistance in nasopharyngeal carcinoma (NPC) [120]. In addition, EBV-LMP1 regulates Drp1 through AMPK and cyclin B1/Cdk1, which promote cell survival and cisplatin resistance in NPC [121].

#### 4.1.4. Fatty Acids and Lipid Metabolism

Human adenovirus type 36 (Ad-36) is known to infect humans and has been reported to be associated with obesity [122]. Ad-36 decreases fatty acid oxidation and increases de novo lipogenesis in primary cultured human skeletal muscle cells by promoting cell death-inducing DNA fragmentation factor-alpha (DFFA)-like effector C/Fat-specific protein 27 (CIDEC/FSP27) expression. Wang et al. reported that AMPK activities were significantly lower in Ad-36-infected cells than in uninfected control cells in both basal and AMP-stimulated conditions. Furthermore, CIDEC/FSP27 siRNA transfection significantly reduced FSP27 expression and partially restored AMPK signaling [123].

Rift Valley fever virus (RVFV) is an enveloped, negative-strand RNA virus of the family Bunyaviridae in the *Phlebovirus* genus. It is a mosquito-borne disease of ruminant animals and humans, which can cause mild to severe symptoms. During RVFV infection, AMPK is activated, leading to the phosphorylation and inhibition of acetyl-CoA carboxylase, leading to decreased fatty acid synthesis and restriction of the RVFV infection [124]. 

HCV also activates PPARα [125] and sterol regulatory element-binding protein-1 (SREBP-1) [126], which increases lipid biogenesis and inhibits mitochondrial β-oxidation. In cells infected with HCV, or harboring an HCV subgenomic replicon, AMPK is significantly inhibited, resulting in enhanced viral replication and lipid accumulation [5]. An in vivo mouse study revealed that ROS-induced activation of AMPK, attenuated de novo lipogenesis and increased β-oxidation, leading to HCV-induced cell cycle arrest [5]. Furthermore, it was shown in vivo and in vitro that HCV nonstructural protein 5A (NS5A) inhibits AMPK phosphorylation. This resulted in increased expression of sterol regulatory element-binding protein-1c (SREBP-1c), acetyl-coenzyme A carboxylase 1 (ACC1) and fatty acid synthase (FASN) and contributed to HCV-associated hepatic steatosis [127,128]. 

In addition, the usage of a direct small molecule activator of AMPK, PF-06409577 inhibited flavivirus infection, including the West Nile virus (WNV), Zika virus (ZIKV), and DENV, through modification of host cell lipid metabolism [9]. 

Activation of AMPK restricts *Coxsackie virus B3* (CVB3) replication by inhibiting lipid accumulation [129]. A recent publication by González-García et al., reported that respiratory syncytial virus (RSV) infection activates AKT-dependent inhibition of AMPK, and induces the activation of downstream lipogenic effectors and cellular lipid anabolism [130]. DENV upregulates the HMG-CoA reductase activity through the impairment of AMPK phosphorylation, and a reduction of AMPK phosphorylation activity was observed in DENV-infected cells at 12 and 24 hpi. Overall, DENV infection increases HMGCR activity through AMPK inactivation leading to higher cholesterol levels in the endoplasmic reticulum, which is necessary for formation of replicative complexes [131,132].

However, there are viruses wherein which AMPK activation contributes to viral replication. In HCMV infection, the virus inhibitory protein, an endoplasmic reticulum-associated, interferon-inducible protein (Viperin), increases AMPK activity, GLUT4, and lipogenic enzyme transcription, and enhances lipid synthesis in HCMV-infected cells. Therefore, HCMV uses viperin, known to be antiviral for other viruses, to modulate the metabolic status of the cell to facilitate its replication [133]. 

Another example is the porcine reproductive and respiratory syndrome virus (PRRSV). PRRSV, a member of the order Nidovirales of the Arteriviridae family, is an enveloped, positive sense, single-stranded RNA virus. PRRSV infection induces the activation of the AMPK-ACC1 pathway and the production of fatty acid synthesis, a process that is essential for viral replication [134].

#### 4.1.5. Glucose Metabolism and Glycolysis

AMPK activation plays an important role in HCV infection. In the setting of glucose concentration reduction in cell culture medium from 4.5 to 1.0 g/L, AMPK is activated along with suppression of HCV replication [11]. When Liraglutide (a long-acting glucagon-like peptide-1 (GLP-1) receptor agonist) is used in HCV infected cells, it activates AMPK, thereby inhibiting HCV replication via an AMPK/TORC2-dependent pathway [135]. HCV has demonstrated direct effects on insulin signaling, which also includes reducing glucose transporter type 4 (GLUT4) expression, increasing gluconeogenic enzymes glucose-6-phosphatase and phosphoenolpyruvate carboxykinase 2 (GC6P and PCK2). HCV nonstructural protein (NSP) 5a was reported to upregulate PEPCK, a key regulator of gluconeogenesis and cellular lipids [136].

JC virus (JCV) is a human neurotropic virus. JCV can cause fatal demyelinating disease, progressive multifocal leukoencephalopathy (PML), and is associated with multiple tumors of the central nervous system. The JCV T-antigen suppresses AMPK activation during glucose deprivation in brain tumor-derived cell lines and exerts control over the cell cycle and glucose metabolic pathways. T-antigen decreases AMPK-dependent G1 cycle arrest, as glucose deprivation induces glycolytic flux and activates the pentose phosphate pathway to maintain ATP production [11].

In Zika infection of human umbilical vein endothelial cells (HUVEC), AMPK restricts the viral replication by potentiating the innate antiviral responses and by inhibiting glycolysis [137]. On the other hand, in HCMV, AMPK activation is beneficial for the virus. It induces the expression of the AMPKα2 catalytic subunit, leading to glycolytic activation and supporting productive viral infection [138]. In CVB3 infection, IFN-β modulates glucose metabolism through a PI3K/Akt-dependent mechanism and decreases the phosphorylation of AMPK. This regulation of glucose metabolism is important for the induction of an effective antiviral response against CVB3 [139].

EBV uses AMPK inactivation to overcome cell growth arrest. EBV-miR-Bart1–5P significantly promotes nasopharyngeal carcinoma (NPC) cell glycolysis and induces angiogenesis in vitro and in vivo. EBV-miR-Bart1–5P directly targets the α1 catalytic subunit of AMPK and consequently regulates the AMPK/mTOR/HIF1 pathway, which impels NPC cell anomalous aerobic glycolysis and angiogenesis. This inhibition of AMPK α1 leads to uncontrolled growth of NPC [140]. It has also been reported that EBV-encoded LMP1 inhibits the LKB1-AMPK pathway to promote proliferation and transformation of human nasopharyngeal epithelial cells [141].

### 4.2. Calcium/Calmodulin Activation 

Human cytomegalovirus (HCMV) grows at a slow rate in tissue culture, allowing viral manipulations to maintain favorable cellular conditions throughout the infection. These conditions activate cellular stress responses including AMPK-mediated inhibition of mTOR kinase. However, this cellular response is circumvented during the immediate-early phase of HCMV infection [142]. Although AMPK is circumvented during the immediate-early phase of HCMV infection, AMPK activity is essential at the immediate early to early transition of viral gene expression. Therefore, it has been suggested that HCMV activates AMPK through CaMKK, and depends on this activation for high titer replication, likely through induction of a metabolic environment conducive to viral replication [143]. Furthermore, human kinome profiling identified AMPK as a requirement during HCMV infection [144].

### 4.3. Regulation of Immune Response

AMPK plays a direct and indirect role in influenza infection. Mint3/Apba3 depletion activates AMPK through IκBα, and Mint3-deficient mice exhibit an improvement influenza pneumonia with a reduced inflammatory response [145]. Likewise, AMPK activators, such as AICAR, reduce excessive inflammation in mice induced by the highly pathogenic influenza virus [146]. Curcumin has a similar effect on inflammation by enhancing IκBα and AMPK [147]. In addition, KSHV infection reduces anti-inflammatory LXA4 secretion to maintain KSHV latency in infected cells. A recent publication showed that in LXA4-treated KSHV-infected cells, host hedgehog signaling was modulated in an AMPK-mTOR-S6 kinase-dependent manner [148]. In VSV infection in mouse cells, AMPK promotes stimulation of interferon genes (STING)-dependent signaling, independent of ULK1 and subsequently promotes cellular innate immunity and antiviral defense [149]. STING is activated by cyclic nucleotides (CDNs) or by host cyclic GMP-AMP synthase (cGAS), which induce a conformational change in STING. The conformational change activates translocation of STING complexed with TANK-binding kinase 1 (TBK1) from ER to the endosomal or lysosomal perinuclear regions. Translocation of TBK1 leads to phosphorylation of interferon regulatory factor 3 (IRF3) and nuclear factor-κB (NF-κB), which translocate from cytoplasm to nucleus and initiate the induction of interferons (IFN) or inflammatory cytokines [150]. 

In addition, collagen deposition is the major cause of myocardial fibrosis related to CVB3 that contributes to impaired cardiac contractile function. AMPK activation inhibits CVB3 related induction of collagen production via the p38 MAPK-dependent pathway in cardiac fibroblasts [151]. Recently, it was reported that the AMPK pathway is involved with the proteasome inhibitor- MG-132 in preventing acute CVB3 myocarditis. MG-132 significantly increases phosphorylation of AMPK, along with reducing levels of proinflammatory cytokines and apoptotic proteins. It also reduces activation of the AKT/NF-κB and ERK pathway [152]. 

Another interesting pathway is of small heterodimer partner (SHP) in the activation of AMPK. SHP is a member of the orphan nuclear receptor family, which is involved in metabolic regulation. Overexpression of SHP in HCV infected cells results in activation of AMPK and reverses HCV profibrogenic features by decreasing TGF-β and fibrotic gene expression [153].

### 4.4. Other Mechanisms of AMPK Regulation

#### 4.4.1. Macropinocytosis and Micropinocytosis

Ebolavirus is in the family of Filoviruses, enveloped viruses containing a negative sense, non-segmented RNA genome. AMPK is required for the macropinocytic internalization of Ebolavirus. AMPKγ gene expression correlates with EBOV transduction and dorsomorphin, a small-molecule and inhibitor of AMPK-inhibited EBOV infection [154]. 

Vaccinia virus, a member of the Poxviridae family, is a large double-stranded DNA virus, which replicates in the host cell cytoplasm. Vaccinia infection activates AMPK, which in turn facilitates vaccinia entry into the host cell thru its ability to modulate the actin cytoskeleton and micropinocytosis [155]. 

#### 4.4.2. Folate Receptor-α

Recently, it was also shown that folic acid inhibits Zika virus by increasing the levels of phosphorylated-AMPK-α, mediated by folate receptor-α (FRα)-AMPK signal transduction [156].

### 4.5. AMPK-Related Plant Viruses

Geminiviruses are a large family of plant-infecting viruses that encode their genetic information on a circular genome of single-stranded (ss) DNA. These viruses are associated with multiple plant diseases such as bright yellow mosaic, yellow mosaic, yellow mottle, leaf curling, stunting, streaks and reduced yields. Geminivirus infection upregulates the expression of two Arabidopsis protein kinases, GRIK1 and GRIK2, which are related to the mammalian AMPK-activating kinases [157].

## 5. AMPK and COVID-19

### 5.1. Background

The first case of the respiratory disease, Coronavirus Disease 2019 (COVID-19), was reported in December 2019 in Wuhan, China. The first death attributed to COVID-19 was identified to be caused by a novel viral strain, severe acute respiratory syndrome Coronavirus 2 (SARS-CoV-2) [158]. Despite increasing efforts to contain the outbreak, the virus rapidly spread across each continent, leading the World Health Organization (WHO) to declare COVID-19 as a global crisis and a pandemic. As of April 2021, the confirmed global cases of COVID-19 have reached 150 million, with a total of 3.2 million confirmed deaths and rising [158]. The emergence of three lethal outbreaks of human Coronaviruses (HCoVs), including the SARS-CoV pandemic in 2003, Middle East Respiratory Syndrome Coronavirus (MERS-CoV) in 2012, and SARS-CoV-2 in 2019, have brought devastating outcomes. SARS-CoV and MERS-CoV were reported to cause lower respiratory tract infection resulting in acute lung injury (ALI), acute respiratory distress syndrome (ARDS), septic shock, and multiorgan failure [159,160]. The rapid viral replication of SARS-CoV-2 triggers a hyperimmune response causing cytokine storm syndrome, which results in respiratory distress, acute ischemic stroke and multi-organ failure [160,161]. The rapid progression of the disease is one of the leading causes of death in patients with COVID-19 [159].

Despite the efforts to expedite vaccine development, the shortage of vaccine availability across the globe and the lack of pharmaceutical interventions or prevention measures have been attributed to the difficulties in containing the pandemic. Elucidating the mechanisms by which SARS-CoV-2 disrupts cellular function and interacts with host factors can help develop potential COVID-19 treatments.

### 5.2. Involvement of Autophagy in SARS-CoV-2

Previous studies have reported evidence that supports the interaction between HCoV infection and autophagy. SARS-CoV infection was reported to increase autophagosome formation in host cells by activating AMPK or inhibiting mTOR leading to downregulation of ACE2 expression [162,163,164]. As previously mentioned, autophagosome formation occurs in phagophore production and expansion. This process commences via the interactions between mTOR kinase and ULK1 protein complex (ULK1/Atg13/FIP200). ULK1 protein complex binds to and activates Beclin 1/Atg6/vsp34 (class III PI3 kinase) protein complex. Activation of vsp34 leads to the formation of phosphatidylinositol 3-phosphate (PtdIns3P), which recruits autophagy-related proteins to phagophore formation and expansion [162,163,164,165]. Cottam et al. demonstrated that autophagosome production by SARS-CoV nonstructural protein 6 (NSP6) located in the endoplasmic reticulum (ER) is necessary for viral replication [166]. The activity of NSP6 depends on Atg5 and vps34 in the ER. They reported that the initial recruitment of autophagy proteins by SARS-CoV NSP6 is dependent upon the increase in PtdIns3P levels [165]. MERS-CoV infection limited autophagy by inducing AKT1-dependent activation of E3-ligase S-phase kinase-associated protein 2 (SKP2), which targets Beclin1 for proteasomal degradation [166].

It is still largely unknown exactly how SARS-CoV-2 affects or manipulates autophagy downstream of AMPK. Recent evidence has suggested that the SARS-CoV-2 viral nucleocapsid and open reading frame (ORF) 8 proteins can indirectly inhibit mTORC1 by interacting with La ribonucleoprotein translational regulator 1 (LARP1) and FK506-binding protein (FKBP) prolyl isomerases 7 (FKBP7), thereby inhibiting autophagy [166,167]. Similarly, Gassen et al. (preprint) reported that autophagy formation is restricted, as SARS-CoV-2 limits glycolysis and protein translation in human bronchial epithelial cells (NCI-H1299) and monkey kidney cells (Vero FM) [168]. Recently published studies have demonstrated a link between the expression of SARS-CoV-2 ORF3a and the formation of autophagosomes. Qu et al. (preprint) demonstrated by measuring microtubule-associated protein light chain 3 (LC3) conversion (a marker of autophagosome formation) that SARS-CoV-2 induces incomplete autophagy in HeLa cells [169]. They reported increased expression of ORF3a following LC3 conversion, suggesting that ORF3a was sufficient to trigger incomplete autophagy. Qu et al. also demonstrated that ORF3a interacts with autophagy regulator, UV-resistant associated gene (UVRAG) by inhibiting the formation of Beclin1/vsp34/UVRAG complex. As a result, autophagosome formation increases, but the fusion of autophagosome and lysosome is impaired [167,169]. Miao et al. also reported that ORF3a sequesters vps39, a component of homotypic fusion and protein sorting (HOPS) complex, which prevents HOPS from interacting with the autophagosomal soluble N-ethylmaleimide–sensitive factor attachment protein receptor (SNARE) complex (STX17/SNAP29/VAMP8), suppressing autophagosome and lysosome fusion [170]. The ability of SARS-CoV-2 ORF3a to interact with the HOPS complex is unique to this strain of coronavirus and not present in SARS-CoV, nor in MERS-CoV infection [164,170]. In conclusion, it can be postulated that SARS-CoV-2 proteins inhibit autophagy or suppress the fusion/maturation of autophagosomes and lysosomes to benefit viral replication. Further studies are needed to examine the downstream pathway targets of AMPK following SARS-CoV-2 infection.

### 5.3. Pharamacological Treatments Targeting COVID-19

Although researchers are focused on developing new vaccines and treatments for COVID-19, simultaneous efforts are made to repurpose established drugs to fight the infection. Researchers have investigated the beneficial use of treating COVID-19 using metformin, a biguanide derivative used to treat type 2 diabetes mellitus (T2DM), due to its antiviral and anti-inflammatory properties [171]. Studies have suggested that metformin activates the AMPK cascade by forming v-ATPase/Ragulator complex, which is associated with LKB1 and axis inhibition protein 1 (AXIN1), thus leading to AMPK activation and inhibition of mTORC1 [172,173]. Other suggested mechanisms of metformin inhibition of SARS-CoV-2 may occur with respect to its interaction with angiotensin converting enzyme 2 receptor (ACE2) following the activation of AMPK. AMPK phosphorylates and enhances the expression of ACE2. Metformin may enhance the phosphorylation of ACE2, leading to a conformational change and decrease binding and entry of SARS-CoV-2 [174,175]. Other mechanisms of metformin action are through AMPK-dependent reduction in lipid storage, increased fatty acid oxidation, and inhibition of glycolysis and lipogenesis [172]. SARS-CoV-2 was reported to increase the production of lipid anabolic enzymes including fatty acid synthase (FASN), a key regulator in palmitate synthesis and acetyl-CoA carboxylase (ACC1), which plays a key role in palmitoylation of proteins by increasing the activity of the PI3K/AKT/mTOR/S6K signaling pathway [172,176]. Activated AMPK blocks fatty acid synthesis directly by inhibiting ACC1, or indirectly by decreasing cellular FASN and ACC1 enzymes via inhibition of mTORC1, or by inhibiting sterol regulatory-element binding protein-1 (SREBP-1) translocation and maturation in the Golgi body, thus preventing lipid gene transcription [172].

Another repurposed treatment to combat SARS-CoV-2 is orlistat, an inhibitor of FASN, commonly prescribed for the treatment of obesity and obesity-related T2DM. Direct inhibition of FASN activity impairs production of palmitate, thereby inhibiting viral protein palmitoylation [172]. Orlistat was also proposed to inhibit VPS34, a phosphoinositide kinase that functions in autophagy, endosomal trafficking, and other cellular functions [177].

Other medicinal compounds used to target SARS-CoV-2 that inhibit activation of the TXNIP/NLRP3 inflammasome by regulating AMPK are curcumin [178] and the nutraceutical berberine [177]. 

**Table 1:** Summary of pathogens and their respective involvement in regulating of AMPK activation, which serves as either beneficial or detrimental to viral replication, growth, and or evasion of host immune responses. (-) indicates undetermined conclusion.

Pathogen	Abbr.	Involvement of AMPK	AMPK Activation Beneficial or Detrimental for Virus	Ref.
Avian Reovirus	ARV	ARV infection upregulates the phosphorylation of AMPK and that AMPK facilitates MKK 3/6 and MAPK p38 signaling.	Beneficial	[89]
		p17 protein of ARV was found to trigger PTEN, AMPK, and PKR/eIF2α signaling pathways to induce autophagy.	Beneficial	[90]
Bluetongue virus	BTV	BTV induces inhibition of the Akt-TSC2-mTOR pathway and upregulation of the AMPK-TSC2-mTOR pathway. Both contribute to autophagy.	Beneficial	[98,99,100]
Coronavirus (COVID-19)	SARS-CoV-2	Viral nucleocapsid and ORF8 interact with LARP1/FKBP7 to inhibit mTORC1, inhibiting autophagy.	Detrimental	[162,163,164,165]
		Limited activation of AMPK decreases glycolysis and protein translation in NCI-H1299 and Vero cells.	Detrimental	[168] preprint
		ORF3a interacts with UVRAG and inhibits formation of theBeclin1/vsp34/UVRAG complex. This impairs fusion of autophagosome and lysosome.	Detrimental	[169] preprint
		ORF3a sequesters vps39, preventing HOPS from interacting with the autophagosomal (SNARE) complex STX17/SNAP29/VAMP8), thus suppressing fusion of autophagosome and lysosome.	Detrimental	[170]
Coxsackie virus B3	CVB3	AMPK activation was reported to inhibit CVB3 related induction of collagen production via the p38 MAPK-dependent pathway.	Detrimental	[151]
		Activation of AMPK restricts CVB3 replication by the inhibition of lipid accumulation.	Detrimental	[129]
		AMPK pathway is involved in the proteasome inhibitor-MG-132 to prevent acute CVB3 myocarditis.	Detrimental	[152]
		IFN-β modulation of glucose metabolism through a PI3K/Akt-dependent mechanism decreases the phosphorylation of AMPK and is important for the effective antiviral response against CVB3.	Beneficial	[139]
		CVB3 induces autophagy via AMPK/MEK/ERK and Ras/Raf/MEK/ERK signaling pathways, which are essential for the life cycle of CVB3.	Beneficial	[88]
Dengue virus	DENV	DENV activates the AMP Kinase mTOR Axis to stimulate a proviral lipophagy, which is essential for its replication.	Beneficial	[129]
		Activation of AMPK, PF-06409577 inhibits dengue virus (DENV), through modification of host cell lipid metabolism.	Detrimental	[9]
		DENV infection at 12 and 24 hpi increases HMGCR activity through AMPK inactivation leading to higher cholesterol levels in the ER as necessary for replicative complexes formation.		[131,132]
Duck Enteritis virus	DEV	DEV induces autophagy via increasing cytosolic Ca2+ leading to activation of AMPK–TSC2–mTOR signaling pathway.	Beneficial	[101,102]
Ebola virus	EBOV	AMPK is required for the macropinocytic internalization of ebolavirus.	Beneficial	[154]
Epstein–Barr virus	EBV	Activation of AMPK elevates autophagy through an increase in the p53 pathway of sestrins and a reduction in mTOR signaling, which prevents cell transformation.	Detrimental	[88]
		EBV-miR-Bart1–5P directly targets the α1 catalytic subunit of AMPK and consequently regulates the AMPK/mTOR/HIF1 pathway, which impels NPC cell anomalous aerobic glycolysis and angiogenesis.	Detrimental	[140]
		EBV-encoded LMP1 inhibits the LKB1-AMPK pathway to promote proliferation and transformation of human nasopharyngeal epithelial cells.	Detrimental	[142]
		EBV-LMP1 is a suppressor of the DNA damage response through DNA-PK/AMPK signaling and promotes radioresistance in NPC	Detrimental	[120]
		EBV-LMP1 regulates Drp1 through AMPK and cyclin B1/Cdk1, which promote cell survival and cisplatin resistance in NPC.	Detrimental	[121]
Geminivirus	-	Geminivirus infection was shown to upregulate the expression of two *Arabidopsis* protein kinases—GRIK1 and GRIK2, which are related to the mammalian AMPK-activating kinases.	Beneficial	[157]
Hepatitis B virus	HBV	HBV exerts an antiapoptotic effect by activating the AMPK/MnSOD signaling pathway mediated by the HBV X protein.	Beneficial	[106]
		HBx activation of both AMPK and mTORC1 in primary rat hepatocytes work as a balancing mechanism to facilitate persistent HBV replication and could also influence HCC development.	Beneficial	[107]
		Low glucose concentration promotes HBV replication by stimulating the AMPK/mTOR-ULK1-autophagy axis.	Beneficial	[78]
		p70 ribosomal S6 kinase (S6K1), a serine/threonine protein kinase, inhibited HBV replication through inhibition of the AMPK-ULK1 pathway and disruption of the acetylation modification of H3K27.	Beneficial	[77]
		MicroRNA-1271 promotes the activation of the AMPK signaling pathway by binding to CCNA1, resulting in the inhibition of the HBV-associated HCC cell HBV-DNA replication, proliferation, migration and invasion, while accelerating apoptosis.	Detrimental	[108]
		PRKAA (a catalytic subunit of AMPK) is activated in response to HBV-induced oxidative stress, which in turn decreases the HBV replication through promotion of autophagic degradation.	Detrimental	[7]
Hepatitis C virus	HCV	In cells infected with HCV or harboring an HCV subgenomic replicon, AMPK was significantly inhibited, resulting in enhanced viral replication and lipid accumulation.	Detrimental	[5]
		AMPK is activated in response glucose reduction and leads to suppression of HCV replication.	Detrimental	[10]
		HCV induces hepatic metabolism disorders through downregulation of the SIRT1–AMPK signaling pathway.	Detrimental	[117]
		Overexpression of SHP in HCV infected cells results in activation of AMPK and reversed HCV profibrogenic features by decreasing TGF-β and fibrotic gene expression.	Detrimental	[153]
		HCV NS5A protein inhibits AMPK phosphorylation. This results in an increased expression of SREBP-1c, ACC1 and FASN, which contributes to HCV-associated hepatic steatosis.	Detrimental	[122]
		ROS-induced activation of AMPK, attenuates DNL and increases β-oxidation, processes that are associated with HCV-induced cell cycle arrest.	Detrimental	[127,128]
		Metformin activates AMPK following activation of type I interferon signaling and subsequently inhibits HCV replication.	Detrimental	[83]
		Liraglutide (GLP-1) receptor agonist, activates AMPK, which inhibits HCV replication via an AMPK/TORC2-dependent pathway.	Detrimental	[135]
Herpes simplex virus Type 1	HSV-1	HSV-1 modulates the AMPK/Sirt1 axis differentially during the course of infection, interfering with proapoptotic signaling and regulating mitochondrial biogenesis.	Detrimental at early stages of infection; Beneficial during later stages	[113]
		Activation of the AMPK/Sirt1 axis with resveratrol and quercetin, significantly increases the viability of infected neurons, and reduces the viral titer and the expression of viral genes	Detrimental	[114]
		TDRD7 inhibits AMP-activated protein kinase and thereby restricts autophagy-independent virus replication.	Beneficial	[84]
Human adenovirus Type 36	Ad-36	Ad-36 inhibits AMPK and decreases fatty acid oxidation and increases de novo lipogenesis promoting Cidec/FSP27 expression.	Detrimental	[123]
Human Cytomegalovirus	HCMV	AMPK-mediated inhibition of mTOR kinase is circumvented during the immediate-early time of human cytomegalovirus infection.	Detrimental	[140]
		HCMV activates AMPK through CaMKK, and depends on this activation for high titer replication, likely through induction of a metabolic environment conducive to viral replication.	Beneficial	[143]
		Human kinome profiling identified AMPK to be required during HCMV infection.	Beneficial	[144]
		Viperin increases AMPK activity resulting in increase of GLUT4 and lipogenic enzyme transcription, and enhances lipid synthesis observed in HCMV-infected cells.	Beneficial	[133]
		HCMV induces the expression of the AMPKα2 catalytic subunit, which leads to glycolytic activation and support of productive viral infection.	Beneficial	[138]
		Digitoxin inhibits the α1 subunit pump-dependent AMPK activation and leads to increased autophagy at a level that inhibits HCMV	Beneficial	[81]
		ULK1 phosphorylates the HCMV tegument protein pp28 and regulates virions release.	Beneficial	[8]
Human immunodeficiency virus Type 1	HIV-1	HIV-1 Tat inhibits the AMPK signaling pathway through the NAD+/SIRT1 pathway and induces HIV-1 LTR transactivation.	Detrimental	[115]
		MiR-217 is involved in Tat-induced HIV-1 LTR transactivation by downregulation of SIRT1.	Detrimental	[116]
		HIV-1 gp120 V3 loop activates the AMPK/mTOR pathway causing excessive autophagy in neurons, which results in neuronal apoptosis.	-	[92]
Influenza A virus	IAV	Mint3/Apba3 depletion activates AMPK through IκBα and Mint3-deficient mice exhibits improved influenza pneumonia with reduced inflammatory.	Detrimental	[145]
		The AMPK activator, AICAR, reduces the excessive inflammation induced by highly pathogenic influenza virus infection in mice.	Detrimental	[146]
		Curcumin enhances IκBα and AMPK, and reduces inflammation	Detrimental	[147]
		Deficiency of HIF-1α enhances influenza A virus replication by the activation of the AMPKα-ULK1 signaling pathway, promoting autophagy in alveolar type II epithelial cells.	Beneficial	[96]
John Cunningham virus (also referred as, Human polyomavirus 2)	JCV	T-antigen suppresses AMPK activation and exerts control over the cell cycle and glucose metabolic pathways.	Detrimental	[11]
Kaposi’s sarcoma associated Herpesvirus	KSHV/ HHV-8	KSHV infection of endothelial cells enhances angiogenesis, activates the PI3K/Akt/mTOR pathway, and inactivates AMPK.	Detrimental	[109]
		KSHV K1 protein promotes cell survival via its association with AMPKγ1 following exposure to stress.	Detrimental	[110]
		Activated AMPK restricts KSHV lytic replication in primary human umbilical vein endothelial cells.	Detrimental	[148]
		KSHV infection reduces anti-inflammatory LXA4 secretion to maintain KSHV latency in infected cells. In LXA4-treated KSHV-infected cells, host hedgehog signaling is modulated in an AMPK-mTOR-S6 kinase-dependent manner.	-	[111]
Newcastle disease virus	NDV	NDV HN and F glycoproteins stimulates AMPK kinase and downstream ULK1 activation to suppress mTORC1 signaling. This results in a steady state autophagy flux that is essential for its replication.	Beneficial	[93]
Porcine circovirus Type 2	PCV-2	During PCV2 infection AMPK and ERK1/2 induce autophagy through inhibition of mTOR by the phosphorylation of TSC2, resulting in enhancement of its replication.	Beneficial	[94]
		PCV2 ORF5 protein is essential for PCV2-induced autophagy by activating the AMPK-ERK1/2-mTOR signaling pathways.	Beneficial	[95]
Porcine reproductive and respiratory syndrome virus	PRRSV	PRRSV infection induces the activation of the AMPK-ACC1 pathway and induces production of fatty acid synthesis, both of which are essential for the virus’ replication.	Beneficial	[134]
Rabies virus	RABV	RABV induces autophagy in human and mouse neuroblastoma cell lines by the activation of the AMPK signaling pathway.	Beneficial	[103]
		RABV infection activates the AMPK-AKT-mTOR and AMPK-MAPK pathways.	Beneficial	[104]
Respiratory syncytial virus (also referred as, human orthopneumovirus)	RSV	RSV infection induces autophagy through reactive oxygen species (ROS) generation and activation of the AMPK-mTOR signaling pathway to promote viral replication.	Beneficial	[87]
		RSV infection activates AKT-dependent inhibition of AMPK, and induces the activation of downstream lipogenic effectors, resulting in cellular lipid anabolism and favoring RSV replication.	Detrimental	[130]
Rift Valley fever virus	RVFV	During RVFV infection, AMPK is activated leading to the phosphorylation and inhibition of acetyl-CoA carboxylase, resulting in decreased fatty acid synthesis and this restricts the RVFV infection.	Detrimental	[124]
Sendai virus	SeV	TDRD7 inhibits AMPK and subsequently inhibits autophagy-independent replication of viruses from the Paramyxoviridae family, including human parainfluenza virus type 3, respiratory syncytial virus and Sendai virus.	Beneficial	[85]
		AMPK phosphorylates Mff, leading to the disorganization of MAVS clusters. and represses the acute antiviral response	Beneficial	[119]
Swine fever virus	CSFV	CAMKK2/CaMKKβ-PRKAA/AMPK axis is involved in CSFV-induced autophagy, favoring viral replication.	Beneficial	[105]
Vaccinia virus	VACV	Vaccinia infection activates AMPK, which in turn facilitates vaccinia entry into the host cells thru its ability to modulate the actin cytoskeleton and micropinocytosis.	Beneficial	[155]
Vesicular stomatitis virus	VSV	AMPK promotes stimulation of interferon genes (STING)-dependent signaling independent of ULK1, and subsequently promotes the cellular innate immunity and antiviral defense.	Detrimental	[149]
		VSV infection results in a decreased expression of miR-33/33* in macrophages leading to activation of AMPK and activation of MAVS, contributing to the antiviral innate immune response.	Detrimental	[118]
West Nile virus	WNV	Activation of AMPK activator, PF-06409577.	Detrimental	[9]
		WNV C protein inhibits autophagy by AMPK degradation.	Detrimental	[97]
Zika virus	ZIKV	Activator of AMPK, PF-06409577 inhibits ZIKV through modification of host cell lipid metabolism.	Detrimental	[9]
		AMPK restricts the viral replication by potentiating the innate antiviral responses and by the inhibition of glycolysis.	Detrimental	[137]
		Folic acid inhibits Zika virus by increasing the levels of phosphorylated-AMPK-α mediated by the folate receptor-α (FRα)-AMPK signal transduction.	Detrimental	[156]
		Zika virus infection of human foreskin fibroblast cells depletes nucleotide triphosphate levels, leading to AMPK phosphorylation and caspase-mediated cell death.	-	[112]

## 6. Conclusions

**Figure 4:**
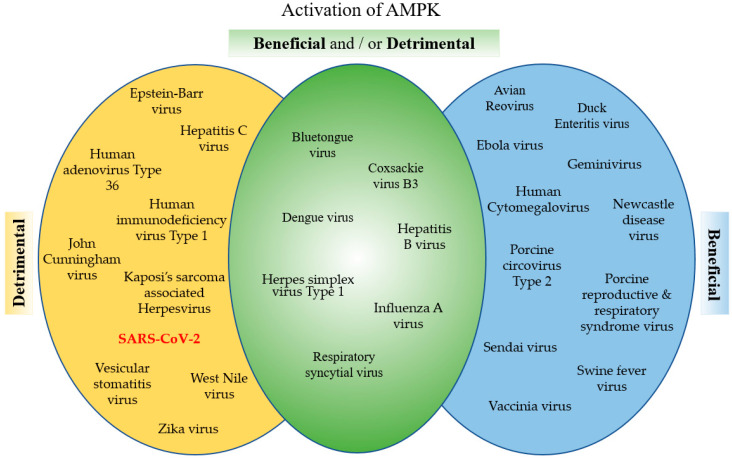
Categorizing viruses based on the effect of AMPK activation. Viral pathogens, sorted based on current literature, concerning whether activation of AMPK is beneficial or detrimental to the pathogens.

AMPK is a powerful metabolic regulator, which maintains cellular energy. AMPK is a key energy regulator of cell growth and proliferation, host autophagy, stress responses, metabolic reprogramming, mitochondrial homeostasis, fatty acid β-oxidation, and host immune regulation function. Due to its important role in cell homeostasis, activated AMPK is an essential cellular factor that many viruses utilize to replicate. On the other hand, activated AMPK enhances the host defenses against viral infection and many viruses have developed mechanisms to inhibit AMPK. Viruses such as HCMV, Ebola and vaccinia virus have developed mechanisms to utilize AMPK activation, favoring their replication. To the contrary, viruses like HCV, HIV and JCV inhibit AMPK to enhance their replication. Interestingly, for some viruses such as DENV, CVB3 and influenza virus, there are contradicting reports about the effect of AMPK on the virus’ life cycle (Figure 4). These differences are dependent upon the type of infected cells or tissues. In addition, AMPK is an important and sensitive regulator balancing cellular energy, and the contradictions may be related to the timing of the observation. Finally, many drugs, such as the AMPK agonists, AICAR and metformin, and the AMPK antagonist, compound C, and drugs that affect the AMPK pathways, demonstrate promising antiviral activity. Recently, it was reported that the FASN inhibitor, orlistat, and the AMPK activator, metformin, inhibit Coronavirus replication and reduce systemic inflammation. These promising findings emphasize the importance of the basic research of the AMPK related pathways for the development and identification of proper and most effective drugs against a specific virus.

## Data Availability

Not-applicable.
